# Endothelium-Independent Relaxation of Vascular Smooth Muscle Induced by Persimmon-Derived Polyphenol Phytocomplex in Rats

**DOI:** 10.3390/nu14010089

**Published:** 2021-12-26

**Authors:** Risa Kudo, Katsuya Yuui, Shogo Kasuda

**Affiliations:** Department of Legal Medicine, Nara Medical University, 840 Shijocho, Kashihara 634-8521, Nara, Japan; yuui@naramed-u.ac.jp (K.Y.); skasuda@naramed-u.ac.jp (S.K.)

**Keywords:** persimmon polyphenols, hyperpolarization, rat, superior mesenteric artery, vascular smooth muscle, vasorelaxation, potassium channels

## Abstract

The vasorelaxant effect of polyphenols is well known, and the mortality rate due to coronary artery disease is low in people who consume polyphenol-containing foods. We aimed to elucidate the mechanism by which polyphenols derived from persimmon juice (PJ) and persimmon leaves (PLs) induce vasorelaxation and suppress vasocontraction in the superior mesenteric arteries isolated from male Sprague Dawley rats. Vasocontraction was induced with 1 µM phenylephrine, and polyphenol-induced vasorelaxation was expressed as a percentage of the previous tone induced by phenylephrine. PJ powder (100 mg/L) induced higher levels of vasorelaxation (mean ± standard error of the mean, 88.6% ± 4.4%) than PLs powder (1 g/L; 72.0% ± 10.8%). Nitric oxide pathway inhibitors (NG-nitro-L-arginine methyl ester + carboxy-PTIO) did not affect persimmon-derived polyphenol-induced vasorelaxation, whereas potassium chloride, tetraethylammonium, and potassium-channel inhibitors did. Vasorelaxation was endothelium independent with both extracts. Phenylephrine-induced vasocontraction was suppressed by pretreatment with PJ and PLs powder, even when inositol triphosphate-mediated Ca^2+^ release and extracellular Ca^2+^ influx were inhibited. These results suggest that persimmon-derived polyphenol phytocomplex cause vasorelaxation and inhibit vasocontraction through hyperpolarization of smooth muscle cells. Persimmon-derived polyphenols may be able to prevent cardiovascular diseases caused by abnormal contraction of blood vessels.

## 1. Introduction

Polyphenols contained in various foods are known to have a wide range of physiological effects, such as antihypertensive, antioxidative, anti-inflammatory, and anti-allergic effects, suppressing carcinogenesis and blood glucose elevation, and ameliorating cardiovascular disease and metabolic abnormalities [1,2,3,4]. There is a negative correlation between the frequency of intake of polyphenol-rich foods (tea, grapes, apples, cocoa, onions, strawberries, etc.) and the risk of cardiovascular disease [5]. In addition, clinical studies conducted on the effects of polyphenols on the circulatory system, revealed that ingestion of polyphenols is effective in lowering blood pressure in patients with mild hypertension [6]. Furthermore, polyphenols ameliorate dyslipidemia and insulin sensitivity [7,8,9,10,11].

Polyphenols are also known to improve the ability of vascular endothelial cells to control the vascular tone and protect the cardiovascular system [12,13]. Polyphenols contained in many foods induce endothelium-dependent vasorelaxation, suppress vasocontraction, and exert antihypertensive effects [14,15]. In experiments with isolated arteries, it has been reported that endothelium-dependent vasorelaxation induced by polyphenols is mainly mediated via the nitric oxide (NO) pathway [16,17,18,19]. Furthermore, in peripheral arteries, polyphenols reportedly cause vasorelaxation via the endothelium-derived hyperpolarizing factor (EDHF) in addition to NO [20,21,22].

The fruits and leaves of persimmons, which are produced in Japan, are rich in polyphenols and were found to exert antihypertensive, antioxidative, anti-inflammatory, and blood glucose-lowering effects [23,24,25]. Regarding vascular function, polyphenols derived from persimmon leaves (PLs) reportedly cause NO-mediated vasorelaxation in conduit arteries such as the aorta [26], but the mechanism of peripheral artery relaxation remains unclear. Furthermore, to the best of our knowledge, there are no studies on the effects of polyphenols derived from persimmon juice (PJ) on peripheral arteries. We hypothesized that PJ- and PLs-derived polyphenols have strong vasorelaxation and vasocontraction inhibitory effects on rat peripheral arteries due to vascular endothelium independence (i.e., vascular smooth muscle hyperpolarization) rather than vascular endothelial cell-derived NO.

## 2. Materials and Methods

### 2.1. Preparation of PJ and PLs Powder

Immature, astringent Japanese persimmon (*Diospyros kaki*) fruits and leaves of the Hiratanenashi cultivar, harvested in Nara Prefecture, were processed to obtain PJ and PLs powder.

The tannin-containing PJ was prepared to obtain the so-called “Kakishibu” powder. The harvested, immature fruits were treated with 0.2% (*v*/*w*) ethanol for 5 days to solubilize the polyphenols. Treated fruits were crushed, immersed in water, and stored at room temperature for 2 days. Thereafter, the supernatant, containing soluble components such as sugar, was removed. Water was added to the residue containing the polyphenols, and the mixture was heated to 120 °C for 30 min to extract the polyphenols. The extract was filtered, evaporated in vacuo, and drum dried at 160 °C to produce a powder. PLs powder was obtained from persimmon leaves that were roasted for sterilization, dried, and pulverized.

The catechin (tannin) concentrations in the PJ and PLs powders were 64.0% and 5.23%, respectively, as measured by using the iron-tartrate colorimetric method [27]. These powders were prepared and provided by Ishii-Bussan, Inc. (Nara, Japan) and stored at −20 °C until use.

### 2.2. Estimation of Catechin Components in PJ and PLs

PJ and PLs powders were dissolved in distilled water (10 g/L), filtered through 0.22 µm membrane filters, and used for further experiments. A Shimadzu 8045 triple quadrupole mass spectrometer with a probe electrospray ionization (PESI) ion source (Shimadzu, Kyoto, Japan) was used for the direct detection of catechin components. The samples were placed directly on a dedicated plastic sample plate and set on the PESI ion source [28]. The probe needle was lowered such that the needle tip touched the sample and then was raised to apply a high voltage for ionization. This vertical movement was repeated, and the generated ions were introduced into the tandem mass spectrometry (MS/MS) system. The PESI-MS/MS conditions were set as follows: probe-applied voltage, 2.3 kV; cycle time for probe movement, 160 ms; desolvation line temperature, 250 °C; heat block temperature, 50 °C; and polarity, negative. The probe position (distance from the tip of the needle to the center of the MS inlet) was set at 2 mm in the y axis and 2.5 mm in the x axis. The product ion (Pl) spectra of the compounds were measured in the multiple reaction monitoring (MRM)-Pl scan mode. The collision energies were adjusted to optimize the product ion signal to be −27, −30, −33, −41 eV for (−) epigallo-catechin-3-gallate (EGCg), (−) epicatechin-3-gallate (ECg), (−) epigallocatechin (EGC), and (−) epicatechin (EC). The MRM mode was used to monitor the transition of the deprotonated molecule at m/z 457.0 → 168.8 (EGCg), 441.0 → 168.8 (ECg), 305.1 → 124.8 (EGC), 289.1 → 108.8 (EC) [29]. The catechin composition ratios were calculated by integrating all peak areas of each compound using the built-in LabSolutions software (ver. 5.99 SP2, Shimadzu Corp., Kyoto, Japan).

### 2.3. Animal Procedures

All protocols for the animal experiments were approved by the Animal Care Committee of Nara Medical University in accordance with the policies established in the NIH Guide for the Care and Use of Laboratory Animals (Permit No. 12689).

Male Sprague Dawley rats (*n* = 20, 10–12 weeks old and weighing 330–350 g; CLEA Japan, Inc., Tokyo, Japan) were placed in a quiet, temperature- and humidity-controlled room, and maintained in a 12 h light–dark cycle (08:00–20:00 light).

### 2.4. Preparation of Rings of Isolated Superior Mesenteric Artery

The rats were euthanized by exsanguination. Their superior mesenteric arteries (SMAs) were excised and the adherent connective tissues were removed. The SMAs were sectioned into rings of 1–1.5 mm in length that were arranged isometrically in vitro, as previously described [30]. Briefly, the rings were horizontally mounted on tension hooks (50 μm in diameter) in 4 mL organ baths containing Krebs–Ringer solution (118 mM NaCl, 4.7 mM KCl, 1.2 mM MgSO_4_, 1.2 mM KH_2_PO_4_, 25 mM NaHCO_3_, 2.5 mM CaCl_2_, and 10 mM D-glucose; pH 7.4). The solution was maintained at 37 °C by using a thermally regulated water circuit, and continuously aerated with 95% O_2_ and 5% CO_2_.

### 2.5. Tension Measurement

SMAs were used in the present study to determine isometric tension, as there is considerable cumulative knowledge of their vascular function. Isometric tension was monitored with a force-displacement transducer (Signal Conditioner/MSC-2, Primetech Co., Tokyo, Japan), connected to one side of each tension hook, and documented with a pen recorder (Pantos Unicorder/U-228, Nihon Kohden Kohgyo Co., Tokyo, Japan). The SMA rings were suspended on the hooks, the tension was set to 0.2 g, and the rings were stabilized in the organ baths at 37 °C for 90 min. The Krebs–Ringer solution was changed every 15 min. The resting tension was maintained at 0.2 g throughout the experiment [31].

For studies of the endothelium-intact vessels, the rings were discarded if acetylcholine-induced relaxation was not ≥80%. For studies of the endothelium-removed vessels, the endothelial cells were removed by rubbing the intimal surface with a stainless-steel wire. The endothelial cell removal was confirmed by the absence of relaxation when the vessel was exposed to 1 µM acetylcholine.

None of the SMA rings were used for repeated measurements; rather, a single dose–response test was performed on each ring. Various inhibitor combinations were tested using different SMA rings isolated from the same rat. Inhibitors were added to the bath 30 min before the addition of phenylephrine.

### 2.6. PJ- and PLs-Induced Vasorelaxation

Relaxant responses were studied in SMAs that had been precontracted with phenylephrine (1 µM). After the contractions reached a plateau, PJ (0.1–100 mg/L) or PLs (0.1–1000 mg/L) powder was cumulatively added to the bath, and concentration–response curves were plotted. Relaxation was expressed as a percentage of the contraction in response to 1 µM phenylephrine.

### 2.7. Effect of PJ and PLs on Potassium Chloride- and Phenylephrine-Induced Vasocontraction

PJ or PLs powder (1 g/L) was added to the bath, and after 30 min, phenylephrine (0.001–10 µM) or potassium chloride (0–80 mM) was added cumulatively. The shrinkage rate was calculated as a percentage of the shrinkage force 25 min after the addition of 60 mM potassium chloride, which was tested in advance without the presence of PJ or PLs powder.

Phenylephrine-induced vasocontraction is triggered via the receptor-operated channel-mediated pathway in smooth muscle cell membranes and the intracellular triphosphate (IP3)-mediated pathway in smooth muscle cells. To clarify which of these pathways were inhibited by PJ and PLs, we investigated the effects of PJ and PLs on phenylephrine-induced vasocontraction by inhibiting each pathway.

### 2.8. Statistical Analyses

All data are expressed as means ± standard error of the mean. The biochemical and physiological parameters were analyzed statistically with one-way analysis of variance followed by Dunnett’s test for comparison of experimental conditions with control conditions. Statistical significance was set at *p* < 0.05.

### 2.9. Chemicals and Drugs

4-Aminopyridine, apamin, carboxy-PTIO, glibenclamide, Nω-nitro-L-arginine methyl ester hydrochloride, (*R*)-(−)-phenylephrine hydrochloride, and TRAM-34 were obtained from Merck KGaA (Darmstadt, Germany). Iberiotoxin, potassium chloride, and tetraethylammonium chloride were obtained from FUJIFILM Wako Pure Chemical Corporation (Osaka, Japan). Xestospongin C (XeC) was obtained from Abcam plc (Cambridge, UK).

## 3. Results

### 3.1. Catechins Contained in PJ and PLs

**Figure 1:**
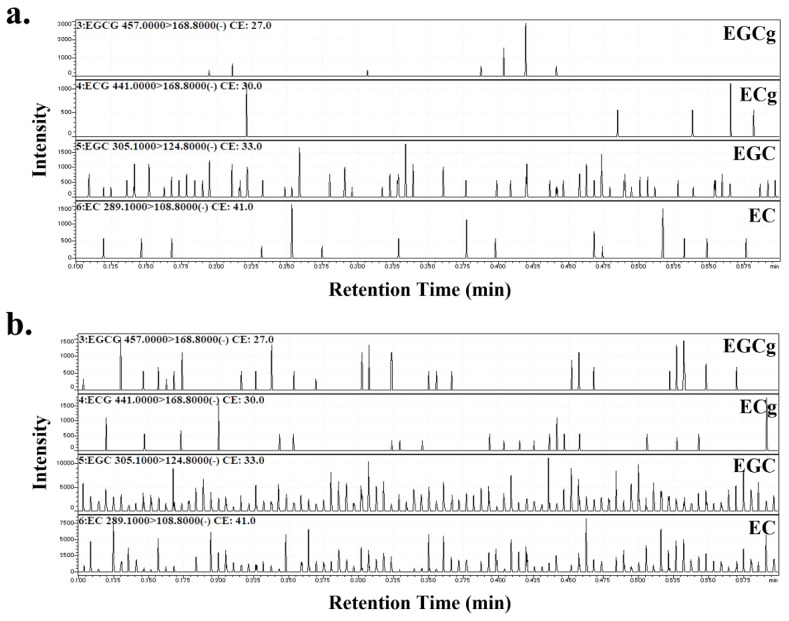
MRM chromatograms of (**a**) persimmon juice (PJ) and (**b**) persimmon leaves (PLs). The product ion (Pl) spectra of the compounds were measured in the multiple reaction monitoring (MRM)-Pl scan mode. The collision energies were adjusted to optimize the product ion signal as −27, −30, −33, −41 eV for (−) epigallo-catechin-3-gallate (EGCg), (−) epicatechin-3-gallate (ECg), (−) epigallocatechin (EGC), and (−) epicatechin (EC). The MRM mode was used to monitor the transition of the deprotonated molecule at m/z 457.0 → 168.8 (EGCg), 441.0 → 168.8 (ECg), 305.1 → 124.8 (EGC), 289.1 → 108.8 (EC).

EC, ECg, EGC, and EGCg, were detected in the PJ and PLs samples. Their composition ratios were EC:ECg:EGC:EGCg = 3:1:11:2 in PJ and 11:1:22:1 in PLs (Figure 1).

### 3.2. Endothelium-Dependent Vasorelaxation Induced by PJ and PLs

**Figure 2:**
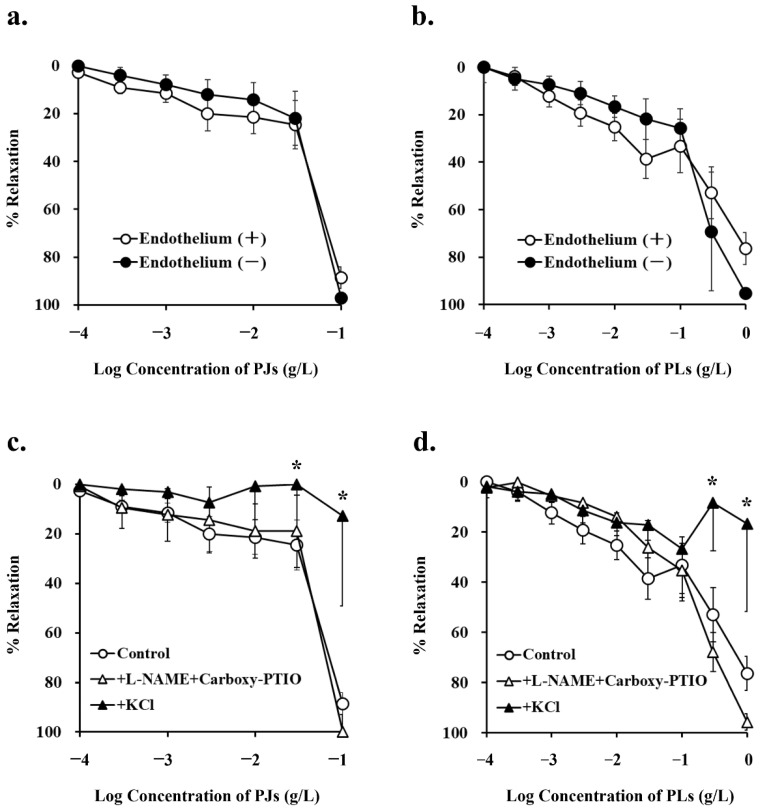
Concentration–response curves for (**a**,**c**) persimmon juice (PJ)- and (**b**,**d**) persimmon leaf (PLs)-induced vasorelaxation in rat superior mesenteric artery rings (*n* = 4–6). (**a**,**b**) These curves were constructed from experiments in the presence and absence of endothelium. (**c**,**d**) These curves were constructed from experiments in the absence (control) and presence of NG-nitro-L-arginine methyl ester (L-NAME; 1 mM) + carboxy-PTIO (0.1 mM) and potassium chloride (20 mM). Results (mean ± standard error of the mean) are expressed as a percentage of the previous tone induced by phenylephrine (1 µM). * *p* < 0.05 vs. the control (Dunnett’s test).

Following precontraction with phenylephrine, PJ and PLs induced dose-dependent relaxation of vessels. Maximum vasorelaxation effects were observed with 100 mg/L PJ powder (88.6% ± 4.4%) and 1 g/L PLs powder (76.4% ± 6.7%). Thus, PJ induced higher levels of vasorelaxation than did PLs. These vasorelaxation effects were not altered by endothelium removal (Figure 2a,b, Table 1).

NG-nitro-L-arginine methyl ester (1 mM) and carboxy-PTIO (0.1 mM), inhibitors of the NO pathway, did not inhibit PJ and PLs-induced vasorelaxation. Potassium chloride, at a concentration that depolarizes cell membranes (20 mM) significantly inhibited PJ- and PLs-induced vasorelaxation (Figure 2c,d, Table 1). These results suggest that vasorelaxation induced by PJ and PLs is caused by hyperpolarization of vascular smooth muscle.

### 3.3. Effect of Potassium-Channel Inhibitors on PJ- and PLs-Induced Vasorelaxation

As endothelial cells were not involved in PJ- and PLs-induced vasorelaxation, we used endothelium-removed vessels to investigate the effects of various potassium-channel inhibitors on PJ- and PLs-induced vasorelaxation at the level of vascular smooth muscle.

**Figure 3:**
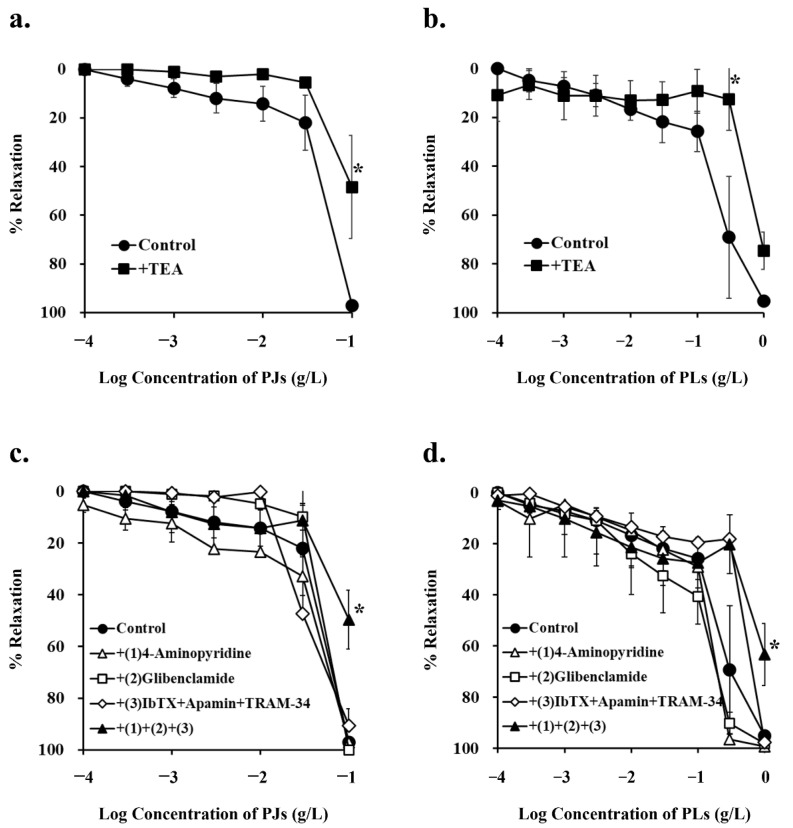
Concentration–response curves for (**a**,**c**) persimmon juice (PJ)- and (**b**,**d**) persimmon leaf (PLs)-induced vasorelaxation in endothelium-removed rat superior mesenteric artery rings (*n* = 4–6). (**a**,**b**) These curves were constructed in the absence (control) and presence of tetraethylammonium (TEA; 1 mM). (**c**,**d**) These curves were constructed in the absence (control) and presence of (1) 4-aminopyridine (1 mM), (2) glibenclamide (10 µM), (3) iberiotoxin (ibTX; 0.1 µM) + apamin (0.1 µM) + TRAM-34 (10 µM), and (1) + (2) + (3). Results (mean ± standard error of mean) are expressed as a percentage of the previous tone induced by phenylephrine (1 µM). * *p* < 0.05 vs. the control (Dunnett’s test).

**Figure 4:**
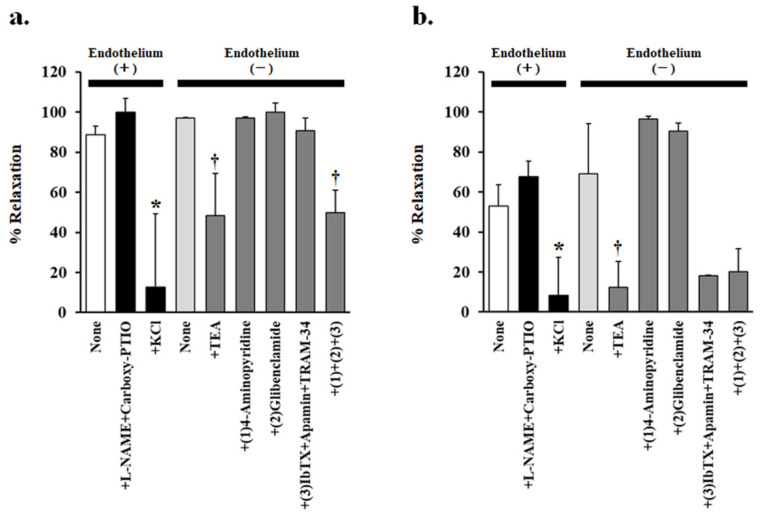
Effects of various inhibitors on (**a**) 0.1 g/L persimmon juice- or (**b**) 0.3 g/L persimmon leaf-induced vasorelaxation in rat superior mesenteric artery rings (*n* = 4–6). The inhibitors added were none, NG-nitro-L-arginine methyl ester (L-NAME 1 mM) + carboxy-PTIO (0.1 mM), potassium chloride (20 mM), tetraethylammonium (TEA 1 mM), (1) 4-aminopyridine (1 mM), (2) glibenclamide (10 µM), (3) iberiotoxin (ibTX 0.1 µM) + apamin (0.1 µM) + TRAM-34 (10 µM), and (1) + (2) + (3). Results (mean ± standard error of mean) are expressed as a percentage of the previous tone induced by phenylephrine (1 µM). * *p* < 0.05 vs. none in the presence of endothelium (Dunnett’s test). † *p* < 0.05 vs. none in the absence of endothelium (Dunnett’s test).

Tetraethylammonium (TEA; 1 mM), a non-selective potassium-channel inhibitor, significantly inhibited PJ (0.1 g/L)- and PLs (0.3 g/L)-induced vasorelaxation (Figure 3a,b and Figure 4a,b, Table 1).

None of the other potassium-channel inhibitors, (1 mM 4-aminopyridine, a potent, non-selective, voltage-gated potassium-channel inhibitor; 10 µM glibenclamide, a vascular, ATP-sensitive potassium-channel inhibitor; 0.1 µM iberiotoxin + 0.1 µM apamin + 10 µM TRAM-34, calcium-activated potassium-channel inhibitors) inhibited PJ-induced vasorelaxation. However, in the presence of a combination of all these inhibitors, PJ (0.1 g/L)- and PLs (1 g/L)-induced relaxation was significantly inhibited (Figure 3c,d and Figure 4a, Table 1).

### 3.4. Effect of PJ and PLs on Potassium Chloride- and Phenylephrine-Induced Vasocontraction

**Figure 5:**
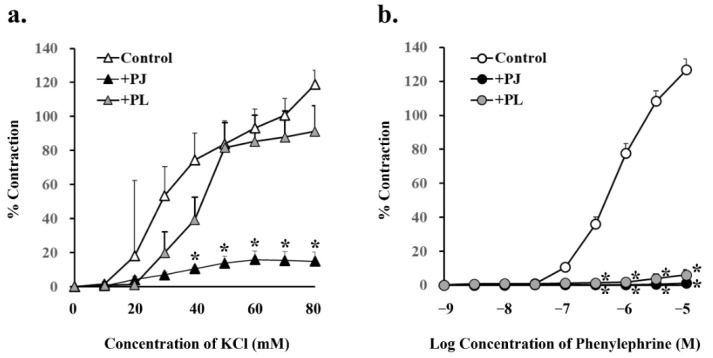
Concentration-response curves for (**a**) potassium chloride- and (**b**) phenylephrine-induced vasocontractions in rat superior mesenteric artery rings (*n* = 4–6). Curves were constructed for experiments with no pretreatment (control) and pretreatment with persimmon juice (PJ) (1 g/L) and persimmon leaf (PLs) (1 g/L) powder. Results (mean ± standard error of mean) are expressed as a percentage of the previous tone induced by potassium chloride (60 mM). * *p* < 0.05 vs. each control (Dunnett’s test).

Concentration-dependent vasocontraction due to potassium chloride via L-type Ca^2+^ channels was significantly suppressed by pretreatment with 1 g/L PJ, but not PLs powder (Figure 5a, Table 2). On the other hand, concentration-dependent vasocontraction due to phenylephrine was significantly suppressed by pretreatment with both PJ and PLs powder (Figure 5b, Table 2).

### 3.5. Effect of PJ and PLs on Phenylephrine-Induced Vasocontraction in the Presence of an Inhibitor of Intracellular Triphosphate-Mediated Ca^2+^ Release and in the Absence of Ca^2+^

**Figure 6:**
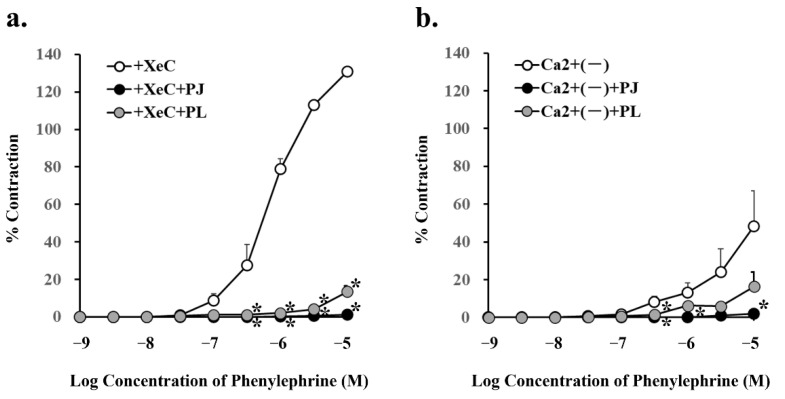
Concentration–response curves for phenylephrine-induced vasocontraction in the absence (control) or presence of pretreatment with persimmon juice (PJ) (1 g/L) and persimmon leaf (PLs) (1 g/L) powder in rat superior mesenteric artery rings (*n* = 4–6). (**a**) The curves were constructed for experiments in the presence of xestospongin C (XeC; 0.5 µM) and (**b**) in the absence of Ca^2+^. Results (mean ± standard error of mean) are expressed as a percentage of the previous tone induced by potassium chloride (60 mM). * *p* < 0.05 vs. each control (Dunnett’s test).

Pretreatment with PJ and PLs powder significantly inhibited phenylephrine (300 nM–10 µM)-induced vasocontraction in the presence of 0.5 µM XeC, a cell-permeable blocker of IP3-mediated Ca^2+^ release (Figure 6a, Table 3).

Phenylephrine (300 nM, 1 µM, and 10 µM)-induced vasocontraction in the absence of Ca^2+^, i.e., with inhibition of the influx of extracellular Ca^2+^ through the receptor-operated Ca^2+^ channels (ROCs), was significantly inhibited by pretreatment with PJ powder. In addition, phenylephrine (300 nM)-induced vasocontraction in the absence of Ca^2+^ was significantly inhibited by pretreatment with PLs powder (Figure 6b, Table 3).

## 4. Discussion

This study demonstrated that PJ- and PLs-derived polyphenol-induced vasorelaxation is not mediated by the endothelial cells, which differs from the results of previous reports. Generally, relaxation in conduit arteries, such as the aorta, is mainly mediated via NO release from the endothelium, and that in the peripheral arteries is mainly via EDHF [32,33]. In fact, many polyphenols reportedly induce NO- and EDHF-mediated relaxation of the endothelium [34,35]. However, in this study, PJ- and PLs-derived polyphenols yielded strong vasorelaxation at the smooth muscle level, but neither of these pathways was found related to this effect.

The effects of potassium-channel inhibitors on vasorelaxation induced by PJ- and PLs-derived polyphenols were non-specific. Therefore, we hypothesize that these polyphenols cause hyperpolarization rather than activation of specific potassium channels. In fact, vasorelaxation induced by PJ- and PLs-derived polyphenols was inhibited by the addition of a depolarizing concentration (20 mM) of potassium chloride, which was further supported by the fact that potassium chloride-induced vasocontraction was suppressed by PJ- and PLs-derived polyphenol pretreatment.

Polyphenols are known to suppress the vasocontraction caused by endothelin-1 and angiotensin II [36,37,38]; however, to the best of our knowledge, this study is the first to demonstrate that PJ- and PLs-derived polyphenols suppress vasocontraction caused by phenylephrine. Pretreatment with PJ- and PLs-derived polyphenols suppressed phenylephrine-induced vasocontraction via both the ROC- and the IP3-mediated pathways. Hence, we hypothesize that these polyphenols cause hyperpolarization of the smooth muscle, thereby reducing the release of Ca^2+^ from the extracellular and intracellular stores of smooth muscle cells into the cytoplasm. In addition, as potassium chloride-induced vasocontraction via L-type Ca^2+^ channels was also suppressed by the PJ powder, it seems that suppression of vasocontraction induced by PJ-derived polyphenols was not receptor-specific.

The phytochemical characterization of the polyphenolic components of the persimmon cultivar used in this study, Hiratanenashi, have already been reported [39,40]. High-performance liquid chromatography and mass spectrum analysis data show that persimmon fruits and leaves possess the catechin polymers, proanthocyanidin, which account for more than 90% of the total polyphenols in these fruits [40] and leaves [41]. Therefore, the vascular effects exerted by PJ and PLs in this study are primarily due to the monomeric and polymeric catechins, with minimal influence from other polyphenols. Moreover, the vascular reactivity of polyphenols derived from PJ was found to be higher than that of the polyphenols derived from PLs. This may, in part, be due to the fact that catechin concentration was more than 10 times higher in the former than in the latter. Second, the difference in the composition ratios of the four catechins EC, ECg, EGC, and EGCg in PJ and PLs may also be pivotal for the superior vascular effects of PJ compared to PLs.

As is frequently reported, all of these catechins have vasorelaxant effects. EGCg reportedly causes vascular endothelium-dependent, NO-mediated relaxation of the rat aorta [42]. EC reportedly causes relaxation in human saphenous veins by activating potassium channels in the smooth muscle [43], a mechanism consistent with the results of this study. In fact, we discovered the same relaxation and contraction-inhibitory effects with standard EGC in preliminary experiments (data not shown) as we did with PJ- and PLs-derived polyphenols. Therefore, the vascular activity of PJ- and PLs-derived polyphenols may be primarily due to the action of monomeric EC and EGC with a high composition ratio in both PJ and PLs. On the other hand, Pu-erh tea reportedly causes vasorelaxation of rat aortas in an endothelium-independent manner, partially via a reduction in the influx of extracellular Ca^2 +^ induced by theabrownins, which are oxidatively polymerized rather than monomeric catechins [44]. Furthermore, PLs contain 40% or more of prodelphinidin, a catechin polymer that reportedly has a vasorelaxant effect [26]. Therefore, the vascular action of PJ and PLs shown in this study may be closely related not only to the monomeric catechins but also to the polymeric catechins produced in the fermentation process. The limitation of this study is that the difference in the structure and composition ratio of the polymeric catechins of PJ and PLs related to vascular action has not been clarified. Further research is needed to clarify the contribution of monomeric and polymeric catechins to the vascular effects of PJ and PLs.

Several experimental and clinical studies have revealed that polyphenols from certain fruits and vegetables reduce blood pressure [45,46,47,48]. As the antihypertensive effects of those polyphenols are mediated via vascular protection at the endothelial cell level [49,50,51], their antihypertensive effects may vary among individuals with cardiovascular disease depending on the degree of endothelial damage. On the other hand, in this study, we revealed that the putative hyperpolarization induced by PJ- and PLs-derived polyphenols acted on smooth muscle cells. Even if the endothelial cells are damaged by hypertension or arteriosclerosis, hyperpolarization will not be affected, which means that these polyphenols may confer a stable antihypertensive effect regardless of endothelial damage.

Furthermore, in this study, PJ- and PLs-derived polyphenols displayed functions similar to those of potassium-channel openers and Ca^2+^ antagonists, both of which are drug types indicated for heart failure. This suggests that persimmons may help ameliorate or prevent hypertension and cardiovascular disease as a functional food. Certain potassium channel openers reportedly reduce the Ca^2+^ sensitivity of contractile elements (Ca^2+^ desensitization) in addition to reducing intracellular Ca^2+^ concentrations via hyperpolarization [52,53]. However, in this study, we did not investigate whether the hyperpolarization of vascular smooth muscle by PJ- and PLs-derived polyphenols reduces the Ca^2+^ sensitivity of contractile elements. Further studies are needed to elucidate the effects of PJ- and PLs-derived polyphenols on Ca^2+^ desensitization in vascular smooth muscle.

## 5. Conclusions

This study showed that PJ- and PLs-derived polyphenols have strong vasorelaxation and vasocontraction-inhibitory effects determined by the hyperpolarization of vascular smooth muscles. As these effects are not mediated via the endothelial release of NO, PJ- and PLs-derived polyphenols may have a preventive effect on cardiovascular events not only in people with normal blood pressure, but also in those with hypertension and endothelial damage.

## Figures and Tables

**Table 1:** Effects of various inhibitors on persimmon juice (PJ)- or persimmon leaf (PLs)-induced vasorelaxation in rat superior mesenteric artery rings.

Inhibitors Added	PJ (0.1 g/L)		PLs (0.3 g/L)	
	% Relaxation	*p* Value	% Relaxation	*p* Value
Endothelium (+)				
none	88.6 ± 4.39		58.4 ± 7.79	
+L-NAME + carboxy-PTIO	100.0 ± 6.99	0.97	72.6 ± 6.58	0.93
+KCl	12.7 ± 36.5 *	0.0002	8.39 ± 19.0 *	0.05
Endothelium (-)				
none	97.1 ± 0.28	0.95 ^1^	69.2 ± 25.0	0.91 ^1^
+TEA	48.4 ± 21.1 ^†^	0.03	12.5 ± 12.9 ^†^	0.04
+(1) 4-Aminopyridine	97.0 ± 0.46	0.85	96.6 ± 1.44	0.99
+(2) Glibenclamide	100.0 ± 4.54	0.89	90.3 ± 4.35	0.98
+(3) IbTX + apamin + TRAM-34	90.7 ± 6.48	0.74	18.2 ± 0.11	0.09
+(1) + (2) + (3)	49.7 ± 11.4 ^†^	0.03	20.2 ± 11.5	0.07

The inhibitors added were none, NG-nitro-L-arginine methyl ester (L-NAME 1 mM) + carboxy-PTIO (0.1 mM), potassium chloride (20 mM), tetraethylammonium (TEA 1 mM), (1) 4-aminopyridine (1 mM), (2) glibenclamide (10 µM), (3) iberiotoxin (ibTX 0.1 µM) + apamin (0.1 µM) + TRAM-34 (10 µM), and (1) + (2) + (3). Relaxations (mean ± standard error of mean) are expressed as a percentage of the previous tone induced by phenylephrine (1 µM). * *p* < 0.05 vs. none in the presence of endothelium (Dunnett’s test). ^†^ *p* < 0.05 vs. none in the absence of endothelium (Dunnett’s test). ^1^ *p* values compared to none in the presence of endothelium. *n* = 4–6.

**Table 2:** Effects of persimmon juice (PJ) and persimmon leaf (PLs) pretreatment on potassium chloride- and phenylephrine-induced vasocontractions in rat superior mesenteric artery rings.

Pretreatment	Potassium Chloride (80 mM)		Phenylephrine (10 µM)	
	% Contraction	*p* Value	% Contraction	*p* Value
Control	118.8 ± 8.46		126.9 ± 6.32	
+PJ (1 g/L)	15.0 ± 5.28 *	0.00002	1.2 ± 0.77 *	0.000001
+PLs (1 g/L)	91.3 ± 15.1	0.10	6.1 ± 3.02 *	0.000001

Contractions (mean ± standard error of mean) are expressed as a percentage of the previous tone induced by potassium chloride (60 mM). * *p* < 0.05 vs. each control (Dunnett’s test). *n* = 4–6.

**Table 3:** Effects of persimmon juice (PJ) and persimmon leaf (PLs) pretreatment on phenylephrine-induced vasocontractions in the presence of xestospongin C (XeC) or in the absence of Ca^2+^ in rat superior mesenteric artery rings.

Condition	Phenylephrine (10 µM)	
	% Contraction	*p* Value
+ XeC (0.5 µM)	130.8 ± 2.32	
+ XeC (0.5 µM) + PJ (1 g/L)	1.2 ± 1.17 *	0.0001
+ XeC (0.5 µM) + PLs (1 g/L)	13.5 ± 2.91 *	0.0002
Ca^2+^ (-)	48.2 ± 18.9	
Ca^2+^ (-) + PJ (1 g/L)	2.1 ± 1.10 *	0.04
Ca^2+^ (-) + PLs (1 g/L)	16.4 ± 7.48	0.14

Contractions (mean ± standard error of mean) are expressed as a percentage of the previous tone induced by potassium chloride (60 mM). * *p* < 0.05 vs. each control (Dunnett’s test). *n* = 4–6.

## Data Availability

The data presented in this study are available on request from the corresponding author.
